# A novel portable device and validation procedure for transcutaneous electrical nerve stimulation

**DOI:** 10.1017/wtc.2025.10004

**Published:** 2025-08-11

**Authors:** Roberto Paolini, Fangqi Liu, Alessia Scarpelli, Andrea Demofonti, Francesca Cordella, Dai Jiang, Andreas Demosthenous, Loredana Zollo

**Affiliations:** 1 Research Unit of Advanced Robotics and Human-Centred Technologies, Università Campus Bio-Medico di Roma, Rome, Italy; 2 Department of Electronic and Electrical Engineering, University College London, London, UK

**Keywords:** closed loop, electric stimulator, sensory feedback, somatotopic stimulation, TENS, wearable stimulator

## Abstract

The adoption of upper limb myoelectric prosthesis is limited by the lack of closed control loop systems. Although the efferent control has already been integrated into these devices, the sensory feedback restoration in the afferent channel still remains an open challenge. Transcutaneous electrical nerve stimulation (TENS) is a promising method for generating somatotopic sensory feedback, allowing the closure of the control loop system. The application of this technique is limited by cumbersome and grid-powered electrical stimulators, making them unsuitable for everyday life, whereas most portable stimulators available on the market are designed for other purposes (e.g., muscular stimulation or pain therapy) and present limited stimulation wave customization. The stimulation devices employed in the literature often produce not fully suitable stimulation parameters and are frequently validated through procedures that do not fully clarify their practical application for sensory feedback restoration. The research aims to present a novel wearable TENS stimulation device (46 g, 62 × 49 × 20 mm) suitable for sensory feedback application. The validation was achieved through a benchtop test and a preliminary analysis on 10 healthy participants comparing the qualities, intensities, and stimulated areas of the sensations elicited by the proposed device and a reference stimulator. The proposed device is capable of delivering charge-balanced stimulation waves over skin-like resistive load and eliciting tingling and vibration sensations with similar intensities compared to the adopted reference.

## 1. Introduction

Upper limb myoelectric prosthetic systems implementing open-loop control have been hardly accepted by prosthesis users, contributing to a device abandonment rate of 19–39% (Svensson et al., 2017). The introduction of sensory feedback allows a closed-loop control strategy, potentially improving the functionality of the prosthetic device, leading the patient to its greater acceptance (Schiefer et al., 2015, 2018; Page et al., 2018; Mastinu et al., 2020).

Several literature studies have been devoted to analyzing somatotopic and nonsomatotopic approaches to elicit sensory feedback. Nonsomatotopic strategies (e.g., vibrotactile [Witteveen et al., 2015] or mechanical [Antfolk et al., 2012; Shehata et al., 2020) elicit sensations in a sensory area of the body unrelated to the quality and the zone of the original sensation. Somatotopic strategies allow the perception of more natural sensations since the stimulus (e.g., electric [Zhang et al., 2015]) applied to the residual limb in correspondence with the residual afferent nerves is directly perceived on the missing hand (Schofield et al., 2014).

Electrical nerve stimulation is an effective solution to drive somatotopic feedback with both invasive and noninvasive approaches. The need for surgical intervention (Navarro et al., 2005) and the limitations of the currently available technological solutions (such as the lack of a completely implantable device for chronic use) make the transcutaneous electrical nerve stimulation (TENS) a granted solution that has obtained promising results to elicit different sensations, such as tactile and pain (D’anna et al., 2017; Osborn et al., 2018; Scarpelli et al., 2024), allowing object stiffness and shape recognition (Vargas et al., 2019a, 2020).

TENS sensory feedback efficacy is strictly related to the adoption of an electrical stimulator capable of eliciting sensations with different qualities and intensities, replicating the interaction with the environment and closing the control loop between the user and the prosthesis. These sensations are generated by modulating a charge-balanced stimulation waveform with adjustable parameters, that is, pulse frequency (PF), pulse amplitude (PA), and pulse width (PW), which also prevent galvanic process that could cause tissue damage (Günter et al., 2019).

Several studies have explored the relationship between fundamental TENS stimulation parameters (i.e., PA, PF, and PW) and the characteristics of the elicited sensations. For instance, Li et al. (2018) adjusted these parameters to modulate tactile intensity and quality in targeted areas of missing limbs, while D’anna et al. (2017) enabled discrimination of force levels, object stiffness, and shapes through a prosthetic limb. Similarly, Vargas et al. (2019b) investigated haptic perceptions induced by simultaneous stimulation of the median and ulnar nerves, and Scarpelli et al. (2020) systematically characterized evoked sensations to replicate slippage by modulating PF, PA, and PW. Nevertheless, these studies primarily relied on commercial benchtop stimulators, which are unsuitable for real-world applications due to their lack of portability.

To enable somatotopic sensory feedback in daily life, wearable stimulators must be developed with high programmability and adherence to TENS parameter requirements. Existing commercial wearable devices, such as InTENSity (RoscoeMedical, 2023), Perfect TENS (TensCare, 2023), R-C101I (Roovjoy, 2023), and Genesy (Globus, 2023), are primarily designed for pain therapy and muscle rehabilitation, offering only predefined stimulation waveforms without real-time customization. In addition, their stimulation ranges and resolutions do not meet the demands of sensory feedback applications.

Wearable TENS research prototypes described in the literature also exhibit limitations. Many studies lack details on stimulation resolution steps (Poletto and Van Doren, 1999; Qu et al., 2011; Karpul et al., 2017; Yang et al., 2020) or present devices with insufficient stimulation capabilities. Moreover, validation phases are often limited, preventing a comprehensive assessment of the device’s effectiveness. Benchtop evaluations frequently employ resistive loads that do not reflect the skin-electrode impedance, compromising the reliability of the results (Wu et al., 2002; Masdar et al., 2012; Wang et al., 2017). Given that the skin-electrode impedance is at least 10 k 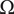 at 500 Hz (Birlea et al., 2014), tests conducted with lower resistive loads should not be considered valid.

Even when appropriate device designs and benchtop tests are performed, preliminary validation on healthy human subjects is often not thoroughly explored. Existing studies primarily assess the ability to discriminate different stimulation patterns rather than investigating the evoked sensations and their localization on the upper limb. For example, Trout et al. (2023) examines discrimination of frequency-modulated signals, while Wang et al. (2021) evaluates the ability to differentiate four distinct modulated stimuli. Table 1 summarizes the stimulation characteristics and validation tests conducted for the main wearable TENS stimulators described in the literature.

**Table 1.** Stimulation characteristics and validation tests of wearable electrical stimulators research prototypes for TENS application

References	PA [mA]	PF [Hz]	PW [ 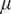 s]	Benchtop test loads	Tests on humans
Wang et al. (2021)	0:0.1:10	1:1:1,000	50:10:450	5,000 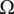	Stimuli recognition
Karpul et al. (2017)	0:–:1	0:–:1,000	N/A	60 k 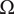 –30 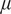 F	N/A
Shirafkan et al. (2020)	0:–:60	1:–:200	100:–:1,000	1 k 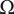 –1 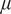 F	N/A
Yang et al. (2020)	0:–:10	N/A	N/A	6 k 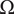 –225 pF	N/A
Poletto and Van Doren (1999)	1:–:25	N/A	25:–:1,000	2,000 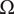	N/A
Trout et al. (2023)	0:–:6	1:–:250	0:–:300	10 k 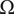	Stimuli recognition
Collu et al. (2023)	0:0.1:25	0:1:500	50:10:10,000	22 k 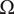 –47 nF	N/A

Abbreviation: N/A, not available.

This work aims to overcome the literature limitations by proposing a novel, wearable, and fully programmable electric stimulator for the upper limb sensory feedback restoration. Furthermore, a novel, systematic protocol is proposed and adopted to evaluate the stimulator performance for restoring sensory feedback.

The validation procedure is divided into two phases: the former is dedicated to bench tests evaluating the error between the desired and measured electrical stimulus; the latter is devoted to preliminary testing of the device on 10 healthy participants, evaluating the elicited areas in the hand and the sensation intensities and qualities. In both phases, the performance of the proposed stimulator was compared with those of a gold standard, that is, the STG4008 (Multichannel System MCS GmbH, Reutlingen, DE), widely adopted in the literature for TENS sensory feedback application (Vargas et al., 2019b; Scarpelli et al., 2020).

This article is organized as follows: Section 2 will include a description of the design requirements and the developed hardware and software components. Therefore, the experimental setup and protocol adopted for the benchtop and preliminary healthy participants’ validations will be detailed. Section 3 will present the main outcomes of both experimental trials. Lastly, Section 4 will discuss the proposed stimulator performance, and it will suggest future developments of the presented work.

## 2. Materials and methods

### 2.1. Design requirements

A comprehensive review of the main mapping studies in the scientific literature was conducted to determine the stimulation characteristics that a TENS device should ensure to effectively elicit somatotopic sensations.

Table 2 presents a comparative analysis of the main mapping studies for upper limb TENS sensory feedback on both healthy and amputee participants. The tested stimulation parameters, including their ranges and modulation steps, as well as key experimental details, such as the type of participants involved, the stimulated limb regions, the surface electrodes used, and the reported outcomes, are listed.

**Table 2.** Comparative analysis of upper limb TENS sensory feedback mapping studies

References	Subjects	Nerves	Electrodes	Stimulator	Parameters modulation	Outcome
D’anna et al. (2017)	4 transradial amputees	Median/ulnar on the forearm	One pair for each nerve ( 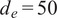 mm)	RehaStim	PA = 2:2: 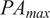 mA PW = 0:20:500 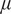 s PF = N-A	Intensity, quality, and elicited zone
Chai et al. (2015)	11 transradial amputees and 8 healthy subjects	Residual limb and the arm	One pair ( 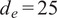 mm)	Master 9	PA = −:0.125:3.75 mA PW = −:20:200 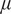 s PF = −:20:50 Hz	Evoked tactile sensation recognition and elicited zone
Zhang et al. (2022)	5 transradial amputees and 14 healthy subjects	Residual limb and the arm	One pair ( 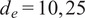 mm)	N-A	PA = 1:0.5:7 mA PW = 20:20:600 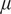 s PF = 10:20:50:100 Hz	Evoked tactile sensation recognition and elicited zone
Shin et al. (2018)	1 transradial amputee and 7 healthy subjects	Residual limb and median nerve	2 × 8 flexible electrode array ( 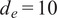 mm)	STG4008	PA = −:0.1:3 mA PW = −:20:200 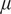 s PF = 150 Hz	Force levels discrimination and elicited zone
Li et al. (2018)	10 transradial amputees	Residual limb	Electrode array ( 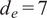 mm)	STG4004	PA = 0.5:0.1:3 mA PW = 20:20:600 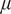 s PF = 25:1:570 Hz	Force levels discrimination and elicited zone
Scarpelli et al. (2020)	9 healthy subjects	Median/ulnar on the forearm	One pair for each nerve ( 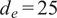 mm)	STG4008	PA = 1:0.1: 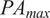 mA PW = 100:40:500 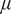 s PF = [50:50:200] Hz PF = [300:100:500] Hz	Intensity, quality, naturalness, depth, pain, and elicited zone
Vargas et al. (2019b)	6 healthy subjects	Median/ulnar on the arm	2 × 8 flexible electrode array ( 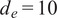 mm)	STG4008	PA = 2:4 mA PW = 200 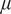 s PF = 150 Hz	Intensity and elicited zone

Abbreviations: 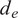 , electrode diameter; 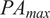 , motor threshold; N/A, not available.

By analyzing these studies, it is possible to define the minimum required parameter ranges and maximum resolution steps necessary to evoke distinct sensations with varying intensities and different perceived locations of somatotopic feedback.

Considering the obtained results of the studies reported in Table 2, suitable variation ranges for eliciting sensations on both healthy and amputee subjects are summarized in the following list:

- PA should range between 0.1 and 3 mA with a step of 0.1 mA;
- PW should range between 20 and 500 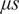 with a step of 20 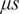 ;
- PF should range between 20 and 500 Hz with a step of 20 Hz.

Despite Zhang et al. (2022) and D’anna et al. (2017) adopting PA values up to 12 mA to elicit tactile somatotopic sensations through the use of grid-powered stimulators (i.e., RehaStim), the authors reckoned that a maximum stimulation current of 3 mA appears to be acceptable since it exceeds the motor threshold (i.e., the maximum current before inducing muscular twitching) for 80% of the participants enrolled in the studies reported in Table 2. According to this, a 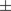 30 V voltage compliance range is sufficient to deliver the desired stimulus to a 10 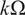 skin-like load. This trade-off allows the development of a restricted power consumption, battery power, and high-energy autonomy device.

The maximum values defined for PF and PW are slightly lower than those tested in the studies by Zhang et al. (2022) and Li et al. (2018). The decision to limit PF and PW to 500 Hz and 500 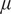 s, respectively, was made because it is possible to elicit the same sensations while delivering a lower charge. This approach enhances both the safety of the stimulation and the energy efficiency of the device. To guarantee the stimulator’s usage in everyday life, the device has to be wearable, battery-powered, and manageable through a wireless connection. The device size and weight should be smaller than those of a modern smartphone to ensure easy wearability, allowing the user to comfortably attach it to the arm using a Velcro strap or place the device in a pocket.

### 2.2. Hardware and software description

This section provides a description of the hardware and software components of the stimulation system, including both the stimulation board and the graphical user interface (GUI) developed for managing the stimulation parameters.

#### 2.2.1. Wearable stimulator design

**Figure 1.**
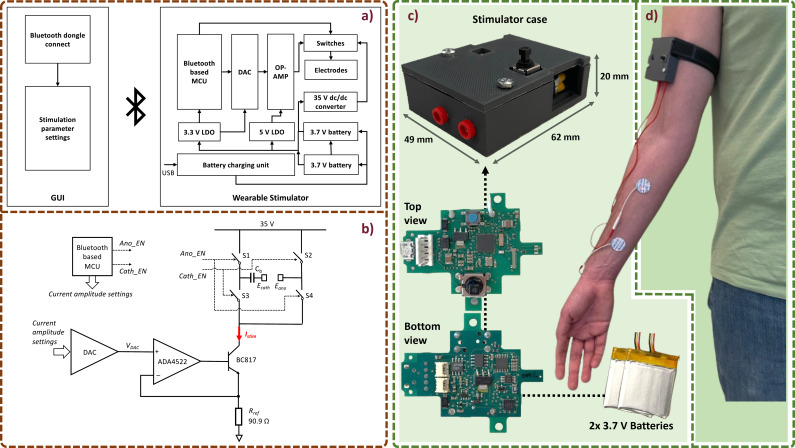
(a) Architecture of the wearable stimulation system, (b) front-end stimulation circuits, (c) wearable case containing the stimulator PCB and two batteries, and (d) image of the stimulation system worn on a participant’s arm.

The overall architecture of the wearable stimulation system is shown in Figure 1(a). It comprises a wearable single-channel electrical stimulator that is programmed and controlled via a MATLAB-based GUI over Bluetooth. The stimulator is powered by two 3.7 V lithium-polymer rechargeable batteries connected in series. The batteries provide a 7.4 V output voltage that is further boosted to 35 V by a DC/DC converter (LM2733YMF, Texas Instruments, USA) for the stimulator output stage. The battery output is also applied to a 5 V low-dropout regulator (LDO) to power the operational amplifier (op-amp) (ADA4522–1 ARMZ, Analog Devices, USA) for generating the stimulation current, and a 3.3 V LDO to power a digital-to-analog converter (DAC) for controlling the stimulation current amplitude and a Bluetooth-based microcontroller unit (MCU) (CC2640R2F, Texas Instruments, USA [Texas Instruments, 2024]) for receiving stimulator settings and controlling the stimulation. The batteries are recharged via a universal serial bus interface.

The schematic of the stimulator output stage is shown in Figure 1(b). The amplitude of the stimulation current, 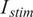 , is controlled by the DAC according to the following relationship(1)

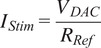

where 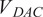 is the DAC output voltage controlled remotely via the Bluetooth link, and 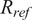 is a 90.9 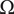 high precision chip resistor (ERJP08F90R9V, Panasonic, Japan). 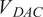 is converted into current by the rail-to-rail op-amp (ADA4522–1ARMZ) and an NPN bipolar transistor BC817.215 (Nexperia, Nijmegen, The Netherlands), forming a unity-gain negative feedback.

The output current, 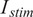 , is converted into biphasic pulses by four switches, S1–S4, in an H-bridge topology (Sacristán-Riquelme and Osés, 2007; Peterchev et al., 2014). Switches S2 and S3 are turned on for the cathodic phase when the control signal 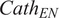 is active, and switches S1 and S4 are turned on for the anodic phase when the control signal 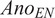 is active. Both 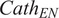 and 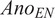 are provided by the MCU. The timing of turning on and off the switches is set remotely through the GUI. Compared to the current source-sink topology, the H-bridge topology is simpler and achieves better charge-balancing performance to keep the electrode–tissue interface within an electrochemically safe limit (Merrill et al., 2005; Williams and Constandinou, 2013). The stimulator has a compliance voltage of 35 V to accommodate a high skin contact impedance. A blocking capacitor, 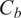 , is connected in series with the electrodes, 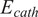 and 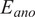 , to prevent DC current through the electrodes in the event of device failure (Nonclercq et al., 2012).

Electrostatic discharge protection circuits were implemented with transient voltage suppression diodes placed between the electrodes and ground. The specifications of the stimulator are detailed in Table 3.

**Table 3.** Stimulator specifications

Parameters	Values
Stimulation current	0.1:0.1:30 mA^a^
Pulse width	20:20:500 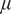 s
Stimulation frequency	20:20:500 Hz
Supply voltage	5 V, 3.3 V
Stimulator output stage voltage	35 V
Battery life	3 h
Battery voltage	2 × 3.7 V
Bluetooth distance	1–3 m

a 1 k 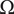 load.

To enhance the wearability of the system, the stimulation board PCB and its power supply batteries were enclosed in a three-dimensionally printed ABS (Acrylonitrile Butadiene Styrene) case, as depicted in Figure 1(c). Figure 1(d) provides an overview of the complete stimulation system worn on a participant’s forearm, highlighting its overall dimensions.

#### 2.2.2. User interface

**Figure 2.**
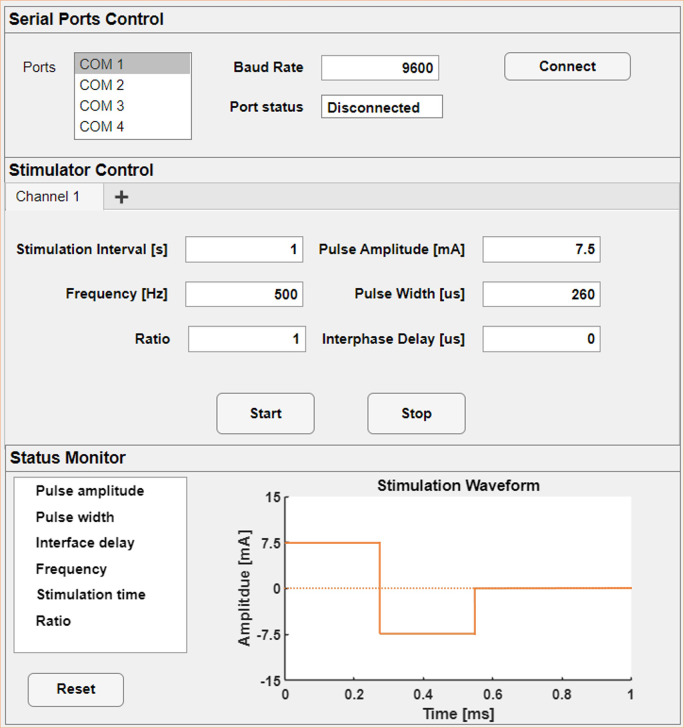
Graphic user interface adopted to manage the bluetooth connection and the real-time stimulation wave customization.

A MATLAB-based GUI was developed using MATLAB R2022b app designer tool (Mathworks, Natick, MA, USA) to control the stimulation parameters, as shown in Figure 2.

The top panel is used to pair the wearable stimulator via a Bluetooth dongle. The middle panel is used to set the stimulation parameter settings, including the amplitude, width, and frequency of the biphasic stimulation current pulses, as well as the interval between the cathodic and anodic phases in a pulse.

In addition, the “stimulation interval” option is used to set the total stimulation time in each trial, and the “ratio” option allows both symmetrical and asymmetrical biphasic pulses by specifying the ratio between the amplitudes of the cathodic and anodic pulses while maintaining charge balance. Upon activating the “Start” button, the stimulation parameters are wirelessly transmitted to the wearable stimulator, which then starts to generate stimulation current outputs.

### 2.3. Benchtop validation

#### 2.3.1. Experimental setup

Bench tests are designed to evaluate the stimulator’s ability to produce the desired waveform. The setup consisted of the developed stimulator and its control GUI, a commercial bench stimulator and its control software, an oscilloscope (Teledyne’s T3DSO1202A 200 MHz model, CA, USA), and five 5% tolerance resistive loads (4.7, 5.6, 6.8, 8.2, and 10 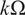 ) (Collu et al., 2023). The bench stimulator adopted as a reference is the STG4008 (Multichannel System MCS GmbH, Reutlingen, DE), a fully programmable device with eight independent stimulation channels. Its proprietary software (MC Stimulus II) allows the total customization of the stimulation wave.

#### 2.3.2. Experimental protocol

To verify the proposed stimulator current range and modulation steps, a series of stimulations were carried out on different resistive loads. After using the oscilloscope to measure the resistor voltage generated by the stimulation current, it was possible to obtain the real stimulation current amplitude and calculate its error compared to the desired one.

Eight PA values were tested on each load with an increasing value from 0.1 to 3.5 mA with a step of 0.5 mA. The PF and PW were set to the maximum values provided by the developed stimulator (i.e., 500 Hz and 500 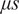 , respectively) and the stimulation duration was set to 1 s. For each delivered stimulus, it was verified there were no offsets and the current was measured.

These tests were made with both the developed stimulator and the reference one. The percentage error between the desired current and the measured one for both stimulators was calculated as follows(2)

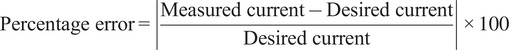



If the error exceeded 5%, it means that the incorrect result was due to the stimulator limitation rather than the load tolerance.

### 2.4. Preliminary healthy participants’ validation

#### 2.4.1. Experimental setup

The purpose of the preliminary healthy volunteers validation was to verify the ability of the developed stimulator to induce different sensations by varying the stimulation parameters and compare the results to those from the reference commercial stimulator.

**Figure 3.**
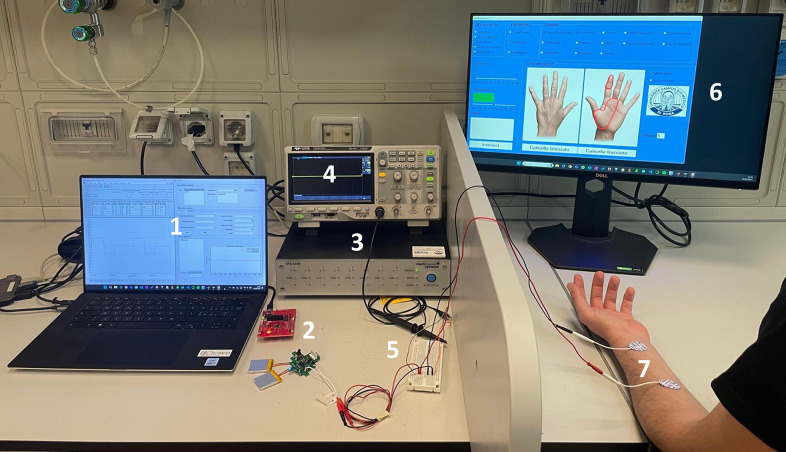
Preliminary healthy participants validation experimental setup. The stimulators could be programmed independently using the two GUIs (1). The proposed device (2) and the STG4008 (3) were alternately connected to a breadboard (5). In the same breadboard, an oscilloscope (4) and the two stimulation electrodes (7) were also connected. Finally, through the GUI on the right screen (6), the participant could describe the elicited sensation.

Ten participants (five men and five women, 28 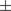 2 years) were enrolled in the study. The main aspects of the study were explained to the participants in a comprehensive language and they signed an informed consent. All the participants had no previous experience with TENS. In Figure 3, the experimental setup is reported.

The participants’ somatotopic sensory responses were elicited by alternate use of both the developed stimulator and the STG4008, as well as their respective control software. The participant was instructed to sit comfortably with his left forearm resting on a table, and two circular (25 mm diameter), commercial, auto-adhesive, and superficial electrodes (TensCare Ltd, Epsom, UK) were placed over it to stimulate the median nerve. The choice of stimulating the left arm rather than the right one has been arbitrary due to the symmetry of the human body with respect to the sagittal plane, whereas the median nerve was chosen over the ulnar one since it innervates the thumb, the index, the medium, and part of the ring finger. The subjects’ skin was not prepared for an even more realistic simulation.

A graphical interface previously developed and used in Scarpelli et al. (2020) was employed to record the outcomes of the participants during the stimulation sessions, allowing them to specify the naturalness, depth, quality, and intensity of the elicited sensation. There were five categories of naturalness, namely unnatural, almost unnatural, likely, almost natural, and natural. The subject had the option to express the depth of the stimulus, whether it was superficial, deep, or perceived in both ways. Regarding the sensation quality, the participant had the option to make a nominal classification of the stimulus by selecting from 13 possible options. Lastly, the perceived stimulus intensity ranged from 0 to 10, respectively, from a no-response stimulus to a maximum one.

In this preliminary validation phase, the developed stimulator was not worn, and a breadboard was used to facilitate the electrode connections. These strategies were implemented to ensure that participants could not distinguish which device was delivering the stimulation.

#### 2.4.2. Experimental protocol

The protocol composed of two phases: a preparatory one followed by the evoked sensations mapping phase. In accordance with the study by Scarpelli et al. (2020), the electrodes were optimally positioned on the participant’s forearm and an electrical stimulus with defined stimulation parameters (PW at 500 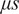 and PF at 500 Hz) was delivered by STG4008. A minimum amplitude of 1 mA was settled, and then it was increased with a step of 0.2 mA until the motor threshold was reached.

Once the subject reported the sensation on the hand rather than above/near the electrodes, the positioning was set, and the sensory range could be defined. This range goes from the sensory threshold (i.e., the lowest amplitude perceived by the subject) to the motor one (i.e., the highest amplitude not producing any muscular contraction). The mapping phase consisted of three preordered modulation sessions, one for each stimulation parameter (PA Modulation, PW Modulation, and PF Modulation).

In particular, for the modulation of PA, with fixed PW and PF respectively at 500 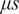 and 500 Hz, the participant was stimulated from the sensitive threshold ( 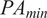 ) to the motor threshold ( 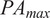 ) with a step of 0.2 mA. The total number of stimulations for each subject varied according to the amplitude of the sensory range.

Subsequently, PW modulation occurred by performing a modulation from 60 to 500 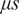 with a step of 60 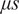 . PF and PA were imposed at 500 Hz and the subject’s motor threshold 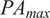 , respectively.

Finally, during PF modulation, the stimulation frequency ranged from 60 to 500 Hz with a 60-Hz step, and imposing the PW and the PA at 500 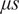 and at 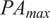 , respectively. During the whole validation, the stimulation duration was kept constant to 1 s.

**Figure 4.**
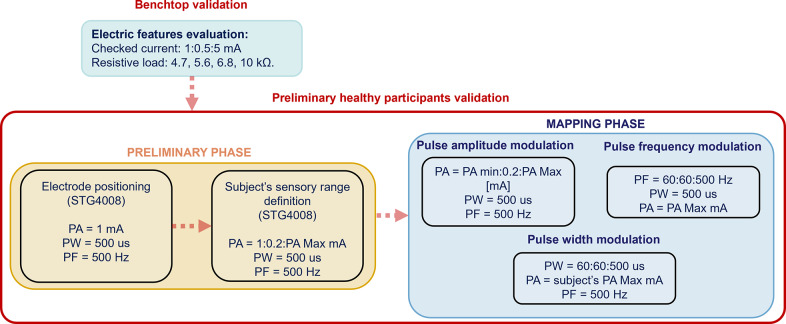
Block diagram showing the validation process for benchtop and healthy participants’ tests.

A schematic representation of the experimental protocol adopted for the benchtop and preliminary healthy volunteers validation, and the relative stimulation parameter ranges of variation are represented in Figure 4. During each parameter modulation, the participant received each stimulus delivered either by the STG4008 or the new stimulator with no awareness of the used device. This was possible through the adoption of an optical barrier located between the volunteer and the hardware components of the experimental setup, and changing the surface electrode connections without moving them on the forearm. After each stimulus, the subject was asked to describe the elicited sensation and its location on the characterization GUI. To prevent the participant from adapting to the stimuli and maintaining high attention, the participant was given 5 min of rest between the three sessions of modulation.

A statistical analysis was conducted on the results obtained from STG4008 and the proposed stimulator during both benchtop and preliminary healthy participants validation, adopting the Wilcoxon rank-sum test. Unless otherwise reported in the figure captions and text, significance level *p* was .05.

## 3. Results

### 3.1. Benchtop validation results

The bench tests validated the stimulator’s electrical performance and compared it to the gold standard (i.e., STG4008). The stimulation wave amplitude analysis, within the first four resistive loads (i.e., 4.7, 5.6, 6.8, and 8.2 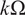 ), demonstrates the efficacy of the proposed device in producing stimulation currents with an average percentage error of 1.46% compared with an error of 0.83% of the reference stimulator.

**Figure 5.**
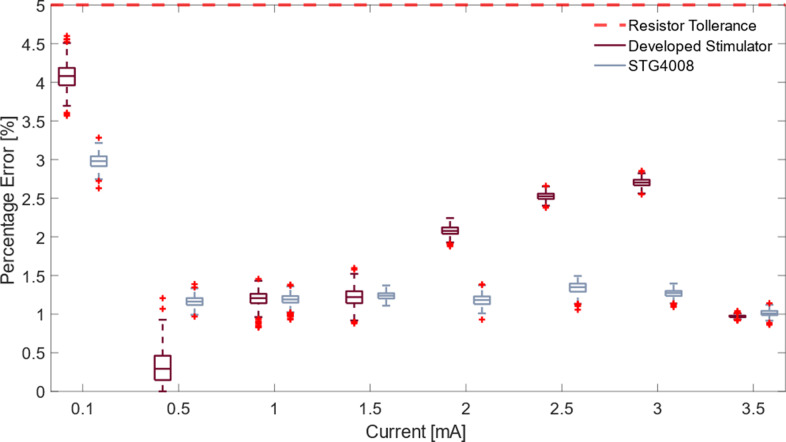
Percentage difference between the produced current and the desired one over a 10 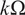 resistive load. Gray and red boxes indicate the errors of the developed device and the reference one, respectively. The red horizontal dashed line indicates the maximum error tolerable. The + signs indicate the outlier.

The current percentage error of the reference and proposed stimulators on 10 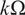 resistive loads are, respectively, represented by red and gray in Figure 5. For the entire amplitude range tested, the reference stimulator produced errors below the maximum threshold.

### 3.2. Preliminary healthy participants’ validation results

The proposed device and the reference one were used to alternately stimulate healthy subjects with somatotopic mapping tests, resulting in three sets of data collection: the intensities of the perceived stimuli, qualitative analysis of evoked sensations, and analysis of elicited zones. In the first two result sets, intensity analysis and quality analysis, the injected charge quantity was calculated to combine PA and PW modulation data.

#### 3.2.1. Perceived intensity

**Figure 6.**
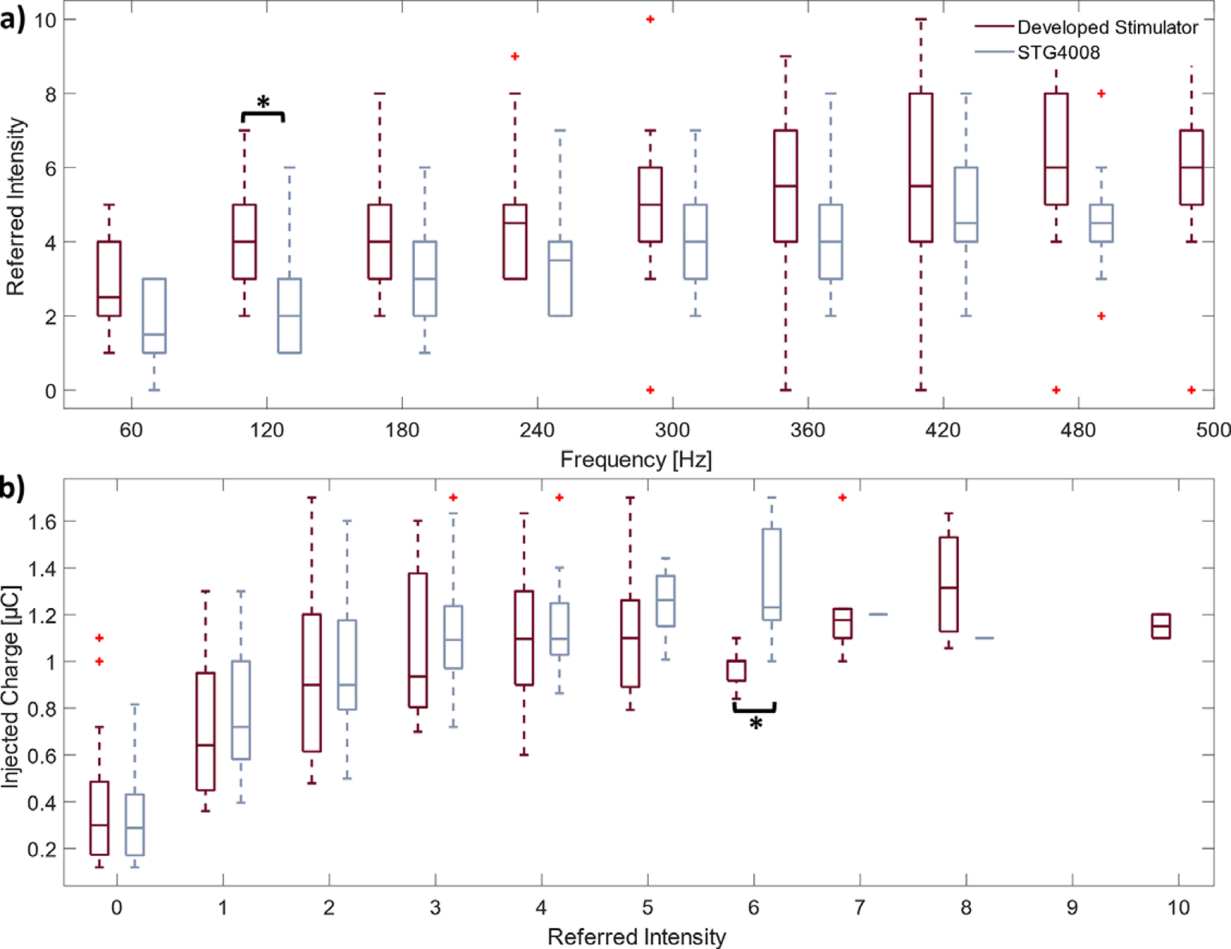
Relationships between the stimulus intensity and the intensity perceived by the participants during frequency modulation (a) and charge modulation (b) performed using the STG4008 (red) and the proposed stimulator (gray). The + sign indicates the outlier. * indicates a statistically significant difference (Wilcoxon rank-sum test, *p* 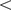 0.05).

The participants reported the intensities of the perceived stimuli on a scale from 0 to 10, and they were collected in the box plots represented in Figure 6. For both stimulators, the perceived intensities increase with an increase in the stimulation frequency.

Although the median values of the intensities indicated by the participants in response to the stimulus delivered by the proposed device are higher than those obtained with the STG4008, the results are similar and there is only a statistically significant difference at 120 Hz (*p* = .04).

The perceived intensity rises as the injected charge increases. The charges needed to produce a stimulus of particular intensity have similar median values. The only statistically significant difference between the two stimulators can be observed at intensity value 6 (*p* = 00007). Lastly, the volunteers did not report an intensity value of 9, while the maximum perceived intensity (10) was only reported using the developed stimulator.

#### 3.2.2. Evoked sensation quality

**Figure 7.**
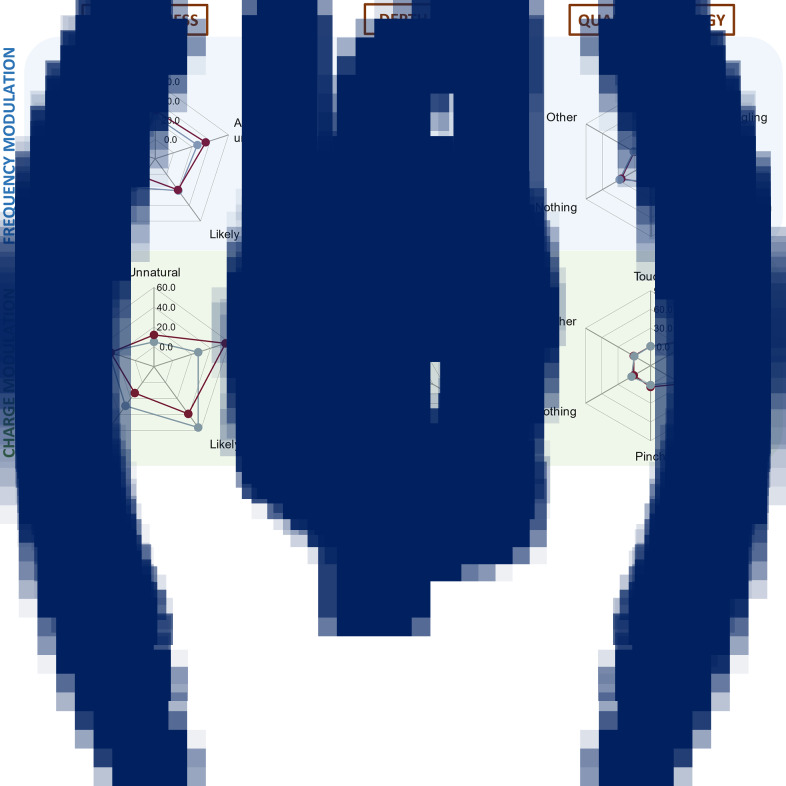
The perceived sensations are described based on three labels: naturalness, depth, and typology. Panels (a--c) report the naturalness, depth, and quality of the elicited sensations during the frequency modulation tests. Panels (d--f) report the same outcomes, in the same order, but for the charge modulation tests. Naturalness has five levels, from unnatural to natural, while depth options include superficial, deep, or both. The top five typologies are reported while the remaining are merged under the “other” item. Colored areas in the plots represent frequency (light blue) and charge (green) modulation.

Figure 7 presents the spider plots indicating the naturalness, depth, and quality of sensations experienced by the participants. The two colored areas bring together the three spider plots that are related to frequency (light blue) and charge (green) modulation.

In the quality typology spider plots, the number of occurrences is almost identical for both modulations. Tingling is the predominant sensation since it represents 61% and 62.5% of the sensations elicited by the presented stimulator during charge (Figure 7(f)) and frequency (Figure 7(c)) modulation, respectively.

The stimulus elicited by the reference has been mostly described as superficial (91% for the frequency modulation in Figure 7(b) and 72% for the charge one in Figure 7(e), while the stimuli elicited through the developed stimulator showed a more homogeneous distribution: in particular, 43, 21, and 36% for “both,” “deep,” and “superficial,” respectively, for the charge modulation.

The STG4008 stimuli have been mostly reported as “almost natural” and “likely” sensations. Conversely, the proposed stimulator elicited more “almost unnatural” sensations: 24 and 38% in frequency (Figure 7(a)) and charge modulations (Figure 7(d)), respectively. Nevertheless, the two stimulator occurrences for the unnatural and natural branches (i.e., the evaluation scale extremes) are comparable. The stimuli elicited by the two stimulators showed statistically significant differences (*p* < .001) in terms of depth and naturalness during both modulations.

#### 3.2.3. Elicited areas

**Figure 8.**
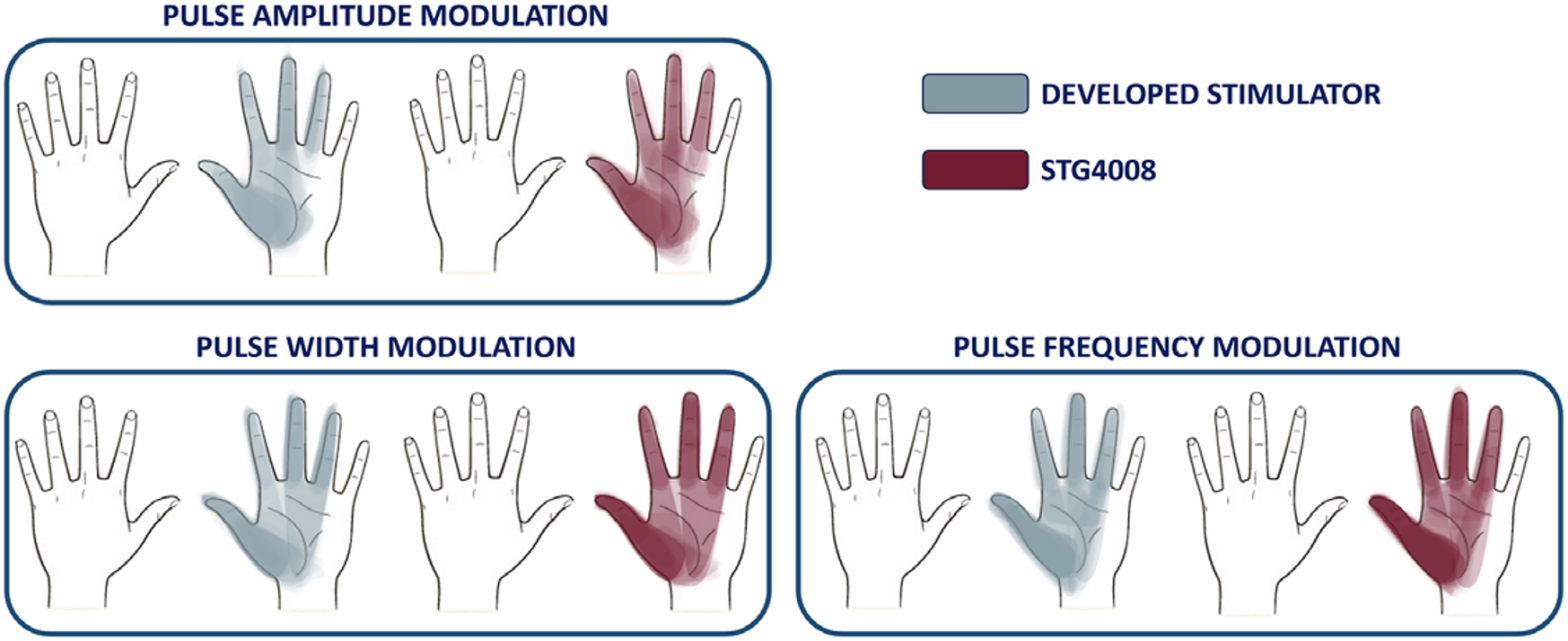
The areas elicited in all the participants by each modulation are reported in the figure. A hand map for each modulation phase is presented.

The volunteers were able to indicate the elicited area of the left hand through a GUI as a result of each stimulus. To create a map, it was necessary to gather all the hands stimulated areas of the 10 participants and overlay them together. Three maps are present in Figure 8, one for each modulation performed. Within each modulation, it is possible to compare the map produced with the reference (red) and the map produced by using the developed device (gray).

During amplitude and PW modulation, the stimulation was mainly perceived in the proximity of the thumb, middle finger, ring finger, and in the lower part of the palm. However, when frequency modulation was used, the stimulated area became more homogeneous, including the index and upper part of the palm. The maps created by the two stimulators in all three modulations are highly similar, both when it comes to the maximum areas stimulated and the areas that are more frequent and darker, that is, the areas where the stimulus was perceived several times.

## 4. Discussion

### 4.1. Design evaluation

The proposed device, including its casing and two 3.7 V batteries, weighs 46 g and measures 62 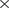 49 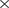 20 mm. These dimensions and mass are comparable to those of other wearable TENS stimulators reported in the literature (Table 4). The compact size and wireless communication make it a wearable device that does not require additional wiring beyond the electrode connections, as shown in Figure 1(d). Given the battery capacity and the device’s maximum power consumption of 740 mW, an operational autonomy of ~3 hr is ensured. For extended experimental sessions, higher-capacity batteries can be employed while maintaining an overall system mass below 150 g, which is less than that of a modern smartphone.

**Table 4.** Comparison of the main wearability characteristics between the proposed device and other literature portable stimulators

References	Dimensions (mm)	Mass (g)	Power consumption (mW)	Battery autonomy (h)
This work	62 × 49 × 20	46	740	3
Wang et al. (2021)	40 × 42 × 26	42	N/A	N/A
Karpul et al. (2017)	48.8 × 26 × 16.9	46	13	19.3
Wang et al. (2017)	90 × 50 × 20	85	720	8
Cornman et al. (2017)	44.8 × 23.6 × 15.4	N/A	900/channel	5

The developed stimulation circuit ensures the delivery of the predefined stimulation parameters, both in terms of range and modulation steps, as established during the design phase. The device voltage compliance of 35 V enables the delivery of stimuli with amplitudes of up to 3.5 mA on a skin-like load, as demonstrated by the bench tests shown in Figure 5. This PA upper limit range was confirmed to be suitable for eliciting somatotopic feedback, as the 3 mA PA design constraint was validated through experimental sessions. Specifically, the participants’ average motor thresholds, assessed using the reference stimulator, were found to be 2.58 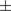 0.4 mA.

### 4.2. Elicited stimuli comparison

The perceived stimulus intensities were found to be stable and scalable across both modulation parameters, in accordance with Vargas et al. (2019b). Specifically, perceived intensities increased with both the injected charge and the stimulation frequency. While the two devices yielded largely consistent outcomes, a statistically significant difference was observed in one instance for each modulation. For frequency modulation (Figure 6(a)), the developed device elicited stronger sensations at the same stimulation frequency (120 Hz). Similarly, for charge modulation (Figure 6(b)), the proposed stimulator achieved the same perceived intensity level (6) while delivering a lower charge. In both cases, the presented system was able to elicit sensations with equal or greater intensity with a lower delivered charge compared to the reference stimulator. This characteristic enhances stimulation comfort while reducing the device’s energy consumption.

Figure 7 illustrates promising results, particularly regarding the type of sensation perceived. Participants primarily reported sensations of tingling or vibration, with no statistical differences between the devices. The predominance of these two sensory qualities is especially evident in frequency modulation, where sensations were perceived as tingling in 62% of cases and as vibration in 30%. In contrast, for charge modulation, this trend was slightly less pronounced, with 61% of outcomes classified as tingling and 20% as vibration. These results are consistent with previous findings in the literature (Zhang et al., 2015; D’anna et al., 2017), which confirms that vibration and tingling are the primary sensations that can be reliably elicited and maintained over time.

The stimuli delivered by the STG4008 were predominantly reported as “superficial” or of intermediate depth in 97% and 90% of cases for charge and frequency modulation, respectively. This result aligns with previous studies on somatotopic feedback, particularly with the findings of Scarpelli et al. (2020), where the same stimulation system elicited sensations classified within these two categories in 90% of cases. In contrast, the proposed device demonstrated a greater ability to modulate the perceived depth of sensation. Notably, in the frequency modulation trials, it was able to elicit sensations perceived exclusively as deep in 21% of the delivered stimuli. This difference highlights the potential for more precise modulation of the depth at which somatotopic stimuli are perceived.

Considering the results of both modulation strategies, the stimuli elicited by the two devices were described as “natural” or “almost natural” in only 24% of cases for the STG4008 and 16% for the developed stimulator. This result confirms that while the sensations elicited through somatotopic feedback are consistent and nonpainful, they still partially evoke the qualities of natural touch (Chai et al., 2015; Shin et al., 2018).

This characteristic is particularly evident for the developed stimulator, which produced sensations more frequently described as “unnatural” or “almost unnatural” compared to the reference device, which primarily elicited sensations described as “likely.”

This difference could be attributed not only to inter-participant variability, but also to the distinct electronic architectures of the two devices. Specifically, the STG4008 employs a passive charge cancellation phase, whereas the presented stimulator actively manages the cathodic phase of the stimulation wave via an H-bridge circuit. This difference could alter the actual charge delivered during stimulation, thereby affecting the characteristics of the elicited sensation. This will be further investigated to determine whether charge cancellation indeed influences elicited sensations.

As shown in Figure 8, both devices stimulated overlapping regions corresponding to the median nerve innervation. While this allows for a direct comparison between the devices, which show the same behavior, the resulting somatotopic map remains specific to this protocol due to the exclusive analysis of healthy participants. In amputees, differences in amputation levels and their neural reorganization can produce more variable and subjective somatotopic maps, whereas in this study, the intact limb resulted in consistent maps across participants.

### 4.3. Limitations and potential future developments

The presented device has undergone a preliminary validation phase that confirmed its ability to elicit somatotopic sensations in healthy participants. While this validation was performed on healthy subjects, the stimulation parameters were designed based on prior studies involving both healthy individuals and amputees. This suggests potential applicability for amputee populations, although further validation is required.

The stimulator was validated in standalone mode. For its effective use, integration with a prosthetic system will be necessary. This would allow stimulation parameters to be modulated in real time by an encoding algorithm, translating the prosthesis–environment interaction into stimulation patterns, eliminating the need for external operator intervention.

Once tested on amputee patients and integrated with a prosthetic system, the device could contribute to further investigating the advantages of somatotopic feedback in prosthetic upper limb control. It would enable participants to dynamically perform activities of daily living (e.g., grasping and handling objects) in unstructured environments, offering a mobility that current TENS stimulators cannot provide.

## 5. Conclusions

In this article, a novel wearable, programmable TENS stimulator designed for the upper limb somatotopic sensory feedback application was proposed. Through the comparison with a commercial reference stimulator, the device was firstly bench tested and then preliminarily validated on a healthy participant, aiming to analyze the quality, intensity, and elicited zones of the evoked sensations. The intensities, qualities, and naturalness of the evoked sensations agree with those reported in the literature studies on somatotopic mapping, and the elicited hand areas are comparable with the areas innervated by the median nerve. Compared to the reference device, the proposed stimulator elicited similar sensations in terms of intensity and quality while offering a greater differentiation in perceived stimulus depth.

Future developments will begin with validating the device in amputees to confirm its effectiveness and establish a maximum current limit. This will help determine whether adjustments to the device voltage compliance are necessary. Next, the device will be integrated into a prosthetic limb to enable closed-loop control. Finally, efforts will focus on adding a second stimulation channel to allow simultaneous activation of both the median and ulnar nerves. This enhancement aims to generate more complex somatotopic tactile sensations, such as slippage, and enable stimulation across the entire hand.

## Data Availability

The datasets generated for this study are available on request to the corresponding author.
